# The relationships between knee extensors/ flexors strength and balance control in elite male soccer players

**DOI:** 10.7717/peerj.12461

**Published:** 2021-11-16

**Authors:** Robert Śliwowski, Jakub Marynowicz, Łukasz Jadczak, Monika Grygorowicz, Paweł Kalinowski, Thierry Paillard

**Affiliations:** 1 Department of Theory and Methodology of Team Sport Games, Poznan University of Physical Education, Poznan, Poland; 2 Department of Physiotherapy, Poznań University of Medical Sciences, Poznan, Poland; 3 Rehasport Clinic FIFA Medical Centre of Excellence, Poznan, Poland; 4 Department of Sport Sciences, University of Pau et des Pays de l’Adour, E2S UPPA, MEPS Laboratory, Tarbes, France

**Keywords:** Quadriceps, Hamstring, H/Q ratio, Total work, Balance score, Isokinetic

## Abstract

**Background:**

Strength and balance are important factors for soccer players to be successful. This study’s aim was to determine the relationship between lower-limb muscle strength and balance control in elite male soccer players (*n* = 77).

**Methods:**

Concentric isokinetic strength (peak torque of quadriceps (PT-Q) and hamstrings (PT-H), hamstrings/quadriceps (H/Q) ratio) was measured for the dominant and non-dominant leg at angular velocities of 60°s^−1^and 240°s^−1^, as well as the total work for extensors (TW-Q) and flexors (TW-H) for both legs (at an angular velocity of 240°s^−1^only). Balance score (BAL score) was used for unilateral assessment of balance control using a Delos Postural System Test measurement tool. Hierarchical multiple regression analyses were performed to predict balance control using isokinetic knee strength performance for dominant and non-dominant legs.

**Results:**

Final modelling included peak torque of hamstrings at 240°s^−1^ and peak torque of the quadriceps at 240°s^−1^ for the non-dominant leg (*R*^2^ = 19.6%; *p* ≤ 0.001) and only peak hamstring torque at 240°s^−1^ for the dominant leg (*R*^2^ = 11.3%; *p* = 0.003) as significant predictors of balance score.

**Conclusion:**

Findings indicate that balance control is widely influenced by peak hamstring torque and peak quadriceps torque at high angular velocity particularly in the non-dominant leg *i.e.,* the supporting leg in soccer players.

## Introduction

Balance control is a fundamental motor skill and can be defined as the individual’s ability to control equilibrium by maintaining or returning the center of body mass over its base of support. Posture corresponds to the position of the different body segments at a given moment, which allows a state of balance of the body. Both the maintenance of the center of mass within the base of support and the modulation of the posture involve the integration of sensory information and the regulation of motor and bio-mechanical processes (Pollock et al., 2000). Balance and posture control play an important role in sports with variable and/or asymmetrical movement patterns (Loeza Magana, Fritzler-Happach & Barrios-González, 2017), such as soccer, which is a sport that involves various multidirectional movements, such as sudden acceleration and deceleration tasks, rapid changes of direction, jumping, and landing (Hamilton et al., 2008; Pau et al., 2015; Hrysomallis, 2011), as well as sport-specific skills, such as kicking, often performed in disturbed balance.

The sensory process includes the somatosensory, visual and vestibular pathways while motor responses are influenced by muscle strength, core stability, coordination, and joint range of motion (ROM) (Hrysomallis, 2011; Blackburn et al., 2000; Bressel et al., 2007; Paillard et al., 2006). The sensory and motor outputs associated with lower extremity injury and performance, have been largely examined in terms of neuromuscular performance (*i.e.,* hip, knee and ankle strength, core stability, lower extremity ROMs) and/or balance performance in different cohorts of athletes. For example, Ruiz-Pérez et al. (2019) demonstrated that dynamic balance control measured using the *Y*-Balance test is widely influenced by, inter alia, hip and knee flexion ROM measures in the sagittal plane, as well as isokinetic knee flexion strength among elite male futsal players. López-Valenciano et al.’s (2019) latest study indicates that passive hip flexion and ankle dorsiflexion with knee flexed ROM measures are also important in the performance of *Y*-balance tests in professional male soccer players. The above studies indicate that balance control performance correlates strongly with a number of factors, including flexibility, strength, and neuromuscular control. Each sport and level of competition require different technical skills and physical capabilities, and regular training sessions cause durable musculo-skeletal and postural adaptations in individual (Bressel et al., 2007; Paillard, 2017).

Sufficient strength of the agonist and antagonist muscles across the joints is needed for good balance during functional activities (Blackburn et al., 2000; Croisier et al., 2008). A small number of reports have been published that explore the relationship between dynamic balance control and muscular strength (especially that of the knee extensors/flexors) in soccer populations. Tasmektepligil (2016) reported a small to moderate correlation between knee extensor-flexor muscular strength (measured from both dominant and non-dominant leg) and balance performance among amateur male soccer players. Lockie et al.’s (2013) study, meanwhile, suggested that amateur team sport athletes (including soccer players) with better dynamic balance—measured by functional reaching in the Star Excursion Balance Test (SEBT)—also tended to exhibit greater muscle strength by producing greater knee extensor torque at the tested isokinetic speeds. Booysen, Gradidge & Watson (2015) reported moderate correlations between eccentric strength and knee extensor power and dynamic balance in a group of university soccer players. In contrast, to date, no relationship has been observed between knee extensor strength and dynamic balance in professional soccer players (*i.e.,* a high level of competition). This was confirmed by López-Valenciano et al. (2019), who did not demonstrate a significant contribution in isokinetic strength of the knee flexors and extensors to the *Y*-Balance test scores for the dominant and non-dominant legs in male and female professional soccer players.

However, there still remains a lack of information about the relationship between knee extensors-flexors strength and balance control among elite soccer players. The varied results from the studies above might in part be due to a number of factors, including sex (male *vs.* female), player age (youth *vs.* adults), and competition level (*i.e.,* amateur *vs.* professional), as well as different methods used for the measurement of the balance control performance and strength values applied in each study. Various tests and measurement methods show diverse strategies and mechanisms for regaining balance control. According to Booysen, Gradidge & Watson (2015) a better understanding of the relationship between neuromuscular qualities and balance control could improve and advance current training interventions (and thus performance) related to their reciprocal influence in professional soccer players.

With this in mind, the purpose of this study was to determine the relationship between lower-limb muscle strength and balance control in elite male soccer players. First, it was hypothesized that lower-limb muscle strength is associated with balance control and, therefore, would be identified as a significant predictor of balance control performance.

Second, it was hypothesized that that the leg’s dominance influence balance performance in unilateral stance—and the non-dominant leg as a stance leg, which supports the body—would present higher level of balance score.

## Methods

### Participants and data collection

Data were collected for 77 field players (26.8 ± 5.4 years; 183.3 ± 6.3 cm; 78,6 ± 7.1 kg) belonging to teams from the top competition level in Poland. All the studied participants were under contract as professional players. All players were informed about the research procedures, requirements, benefits, and risks, and their written consent was obtained before the study began. The study was conducted according to the requirements of the Declaration of Helsinki and the ethical approval for the study was granted by the Bioethical Committee at the Poznań University of Medical Sciences (629/13). Parental or guardian consent was gathered for participants under 18. The study took place from 2010 to 2019. All measurements were carried out twice a year at the Rehasport Clinic FIFA Medical Centre of Excellence in Poznań: first in January/February (at the beginning of the pre-season period in Poland) and then in June/ July, with the season officially starting in August each year. The players had no previous history of major or moderate injury in knee and ankle joints. These data were taken during routine biomechanical prospective evaluations. The measurements were performed by the same team of examiners.

### Test procedures

#### Lower-limb muscle strength testing

Isokinetic knee muscle strength performance data were collected as previously described in Śliwowski et al. (2017) and Śliwowski et al. (2021).

#### Assessment of balance control

Balance control tests were performed using the Delos Postural Proprioceptive System (Delos, Turin, Italy). This system includes the Delos Vertical Controler (DVC) electronic postural reader, Delos Postural Assistant (DPA) electronic postural reader, Delos Equilibrium Board (DEB), and the Postural System Manager (PSM; a computer software package for visualization and analysis of DVC and DEB data). The electronic postural reader device (DVC)—oval-shaped (7 × 4.5 × 2.5 cm in size) and applied to the sternum—measured the trunk inclination in the frontal (x) and sagittal plane (y) by means of a 2-dimensional accelerometer unit. The DPA is a supporting bar with an infrared sensor that detects when touched for support. The electronic rocking board (DEB) had a single degree of freedom on the frontal plane (range of motion: ±15°) and measured the inclination of its moving plate. The rocking board is equipped with an accelerometer connected wirelessly (Bluetooth) to a computer. The data from the postural reader are a stream of acceleration samples taken by converting the sensor outputs into digital data (Riva et al., 2016). The output signal is sampled at 100 Hz. The analyzed output signals are the inclination angles of the rocking board, as a function of time, and the related direction of the inclination. The maximum width of inclination is within a 30° angle (*i.e.,* −15° ≤ α ≤ 15°). The requested accuracy is 0.5°. Postural instability (PI) is derived from the average instability in the frontal and sagittal planes. PIxy, expressed in degrees, is an indicator of the average amplitude of the postural cone of instability (Riva et al., 2016). Board instability (BI) indicates the average inclination (in degrees) of the board in relation to the horizontal plane. Balance control performance (*i.e.,* balance score) is globally derived from the sum of PIxy and BI and is expressed in degrees; the smaller the sum, the better the performance. An unstable platform, designed to permit only lateral movements was used. The subjects were asked to minimize postural instability while in single leg stance on the electronic rocking board. First, the subject performed four trials, two on each leg, alternating with hands alongside the body. The subsequent four repetitions required the subject’s arms to be held behind their back to minimize the attempts to maintain stability using the upper limbs. Each trial lasted 30 s, with a 20-second break after each trial. The break between series was 60-seconds.

### Statistical analysis

All statistical analyses were performed using Statistica Version 13.0 (StatSoft Polska Sp. z o.o. 2020). Descriptive data are presented as means and standard-deviations, whereas percentage difference in the variables between dominant and non-dominant leg is expressed as an absolute value of mean with 95% confidence limits. The normality of each variable was initially tested with the Shapiro–Wilk test, and the coefficients of asymmetry and kurtosis were also ascertained. Depending on distribution of data, a paired *t* test or Wilcoxon test was applied to examine differences between the scores of the dominant and non-dominant limbs for the all neuromuscular variables and balance performance. Likewise, either Pearson or Spearman correlation coefficients were used to assess the relationships between the balance score and the lower-extremity strength measures. To verify the study hypotheses, two separate hierarchical multiple regression models were built, with balance scores as dependent variables for dominant and non-dominant leg. Models included only the independent variables that turned out to be significantly associated with the balance score on univariate analyses. Cohen’s *d* was determined as a measure of effect size for between legs comparisons. Cohen’s *d* lower than 0.2 was considered irrelevant, between 0.2 and 0.49 was small, between 0.50 and 0.8 was considered medium, and greater more than 0.8 was considered high (Cohen, 1988). An alpha level of 0.05 was used for all analyses.

## Results

### Performance characteristics of balance score and isokinetic strength variables for the dominant and non-dominant leg

Descriptive statistics for each variable are displayed in Table 1. No significant differences were observed between legs for the balance score. With regard to isokinetic strength performance variables, significant bilateral strength differences were observed in terms of peak torque of quadriceps at 60° s^−1^ and 240° s^−1^ (*P* = 0.001, *d* = 0.30 and *P* = 0.003, *d* = 0.23; respectively), peak torque of hamstrings at 60° s^−1^ and 240° s^−1^ (*P* ≤ 0.001, *d* = 0.38 and *P* ≤ 0.001, *d* = 0.41), H/Q ratio at 60° s^−1^ (*P* = 0.038, *d* = 0.22), total work of quadriceps at 240° s^−1^ (*P* = 0.015, *d* = 0.19), and total work of hamstrings at 240° s^−1^ (*P* = 0.021, *d* = 0.13), where the dominant leg was significantly stronger than the non-dominant. No significant differences were noted between legs for H/Q ratio at 240° s^−1^.

**Table 1:** Characteristics of balance score and isokinetic strength variables for dominant and non-dominant leg.

**Variables**	**Dominant** **leg**			**Non-dominant** **leg**		Δ% (95% CL)
	Mean ± SD					
**Balance control**						
**BAL score** [degrees]		11.34 ± 2.67	11.29 ± 2.47		9.56 (7.94–11.17)	
**Isokinetic variables of angular velocity at 60** **° s** ^−1^						
PT-Q^*^ [Nm kg^−1^]	3.19 ± 0.37			3.07 ± 0.43		7.93 (6.39–9.47)
PT-H^*^ [Nm kg^−1^]	1.90 ± 0.31			1.76 ± 0.27		10.04 (8.40–11.67)
H/Q^*^ Ratio	0.60 ± 0.09			0.58 ± 0.09		10.27 (8.50–12.04)
**Isokinetic variables of angular velocity at 240** **° s** ^−1^						
PT-Q^*^ [Nm kg^−1^]	2.06 ± 0.25			2.00 ± 0.28		7.32 (5.96–8.68)
PT-H^*^ [Nm kg^−1^]	1.36 ± 0.21			1.28 ± 0.19		8.66 (7.48–9.84)
**H/Q** Ratio	0.66 ± 0.08			0.65 ± 0.10		9.34 (7.91–10.77)
TW-Q^*^ [J kg^−1^]	52.05 ± 7.39			50.72 ± 6.94		7.46 (6.16–8.77)
TW-H^*^ [J kg^−1^]	32.29 ± 6.92			31.38 ± 6.82		8.46 (7.10–9.82)

**Notes.**

* Significant differences between legs (*P* < 0.05).

PT-Q peak torque of the quadriceps PT-H peak torque of the hamstrings H/Q hamstrings/quadriceps ratio TW-Q total work of the quadriceps TW-H total work of the hamstrings BAL score balance score

### Relationships between balance score and isokinetic strength variables

The correlations between the balance score and isokinetic strength variables for the dominant and non-dominant leg are presented in Table 2. For the dominant leg, a significant correlation was observed between the balance score and peak torque of the hamstrings at 240° s^−1^ (r = −0.34, *P* = 0.003), and total work of the hamstrings at 240° s^−1^ (r = −0.31, *P* = 0.006). Conversely, for the non-dominant leg, a significant correlation was found between the balance score and peak torque of the quadriceps at 60° s^−1^ and 240° s^−1^ (r = −0.24, *P* = 0.037 and r = −0.36, *P* = 0.001; respectively), peak torque of the hamstrings at 60° s^−1^ and 240° s^−1^ (r = −0.26, *P* = 0.021 and r = −0.40, *P* ≤ 0.001; respectively), and total work of the hamstrings at 240° s^−1^ (r = −0.24, *P* = 0.035) (Table 2).

**Table 2:** Correlations of balance score with isokinetic strength variables for dominant and non-dominant leg at angular velocities of 60 s^−1^ and 240 s^−1^.

**Variables**	Angular Velocity at 60° s^−1^		Angular Velocity at 240° s^−1^	
	**DL**	**NDL**	**DL**	**NDL**
	*r* *P-* value			
**PT-Q** [Nm kg^−1^]	−0.03 0.770	−0.24^*^ 0.037	−0.18 0.117	−0.36^*^ 0.001
**PT-H** [Nm kg^−1^]	−0.15 0.179	−0.26^*^ 0.021	−0.34^*^ 0.003	−0.40^*^ >0.001
**H/Q** Ratio	−0.14 0.228	−0.08 0.492	−0.22 0.058	−0.06 0.583
**TW-Q** [J kg^−1^]	–	–	−0.11 0.327	−0.16 0.152
**TW-H** [J kg^−1^]	–	–	−0.31^*^ 0.006	−0.24^*^ 0.035

**Notes.**

* Significant correlations (*P* < 0.05).

PT-Q peak torque of the quadriceps PT-H peak torque of the hamstrings H/Q hamstrings/quadriceps ratio TW-Q total work of the quadriceps TW-H total work of the hamstrings

### Predictors of balance performance for dominant and non-dominant leg: hierarchical regression model

**Figure 1:**
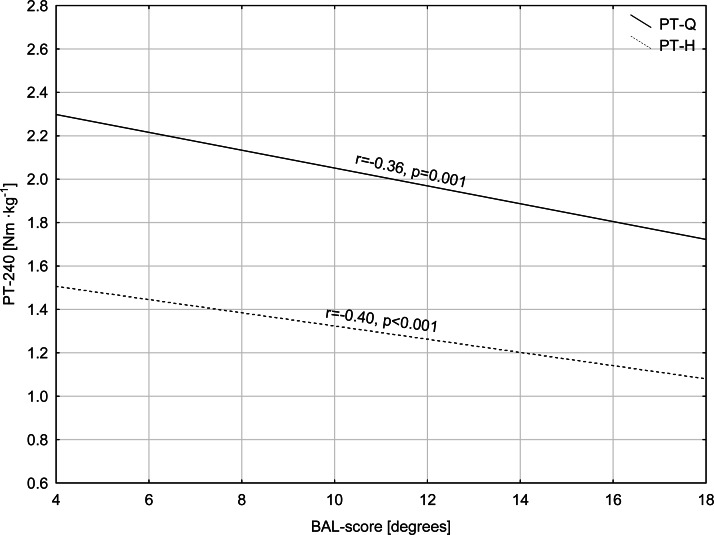
Relationships between peak torque of the quadriceps and hamstrings at 240° s^−1^ and balance score in the non-dominant leg. Note: PT-240, peak torque at 240° s^−1^; PT-Q, peak torque of the quadriceps; PT-H, peak torque of the hamstrings; and BAL score, balance score.

A hierarchical multiple regression was conducted, with the balance score as a dependent variable (Table 3). The model derived for the non-dominant leg postural priority score showed a greater degree of explained variance (*R*^2^ = 19.6%; F (2, 74) = 9.04; *p* ≤ 0.001) than for the dominant leg (*R*^2^ = 11.3%; F (1, 75) = 9.52; p **=** 0.003). Peak torque of hamstrings at 240° s^−1^ and peak torque of the quadriceps at 240° s^−1^ showed a significant association with the balance score for the non-dominant leg (Fig. 1). For the dominant leg, only peak hamstring torque at 240° s^−1^ demonstrated a significant association with the balance score (Table 3).

**Table 3:** Hierarchical multiple regression model for balance scores as dependent variables.

**Variables**		**Explained variance (R2)**			**Regression equation**
		**Model**	**1st** **variable**	**2nd variable**	
**BAL score** [degrees]	**DL**	11.3%	PT-H-240		*Y* = 17.29 − 4.38 x PT-H-240
			11.3%		
**BAL score** [degrees]	**NDL **	19.6%	PT-H-240	PT-Q-240	*Y* = 20.09 − 3.79 x PT-H-240−1.96 x PT-Q-240
			15.7%	3.9%	

**Notes.**

Y composite normalized reach scores obtained from Delos Riva test PT-H-240 peak torque of the hamstrings at 240° s^−1^ PT-Q-240 peak torque of the quadriceps at 240° s^−1^ BAL score balance score

## Discussion

The purpose of this study was to explore the relationships between isokinetic knee muscle strength and balance control in elite male soccer players. The first hypothesis was confirmed as the results showed that lower-limb muscle strength is associated with balance control and could thus be identified as a significant predictor of balance control performance. However, the second hypothesis was invalidated due to the fact that the results did not indicate an influence of leg dominance on balance performance.

Our findings demonstrate that the values of knee extensor-flexor strength are significantly associated to the balance score. In addition, we found that for the non-dominant leg, the value of knee muscle strength significantly predicts the performance of monopedal balance control. The final model included peak hamstring torque at 240° s^−1^ and peak quadriceps torque at 240° s^−1^ for the non-dominant leg, and only peak hamstrings torque at 240° s^−1^ for the dominant leg as significant predictors of balance control performance. It should be emphasized, that peak hamstring torque at 240° s^−1^ was the largest contributor in both the dominant and non-dominant leg, but the magnitude of this effect was greater in the non-dominant leg (see Table 3). To the best of our knowledge, this is the first study which has investigated the association between isokinetic strength measures and balance control measures with the use of the Delos Test in elite male soccer players. The dearth of similar studies in this area—both with regard to playing level of the studied players and study protocol—forces us to make comparisons with footballers playing at lower levels, as well as other athletes, non-athletes and clinical data, in order to discuss the present findings. Caution is therefore suggested in extrapolating the results to other cohorts and different methods of assessing balance control.

Previous research suggested that isokinetic strength of the knee flexors can play an important role in balance control performance. For example Çinar-Medeni et al. (2016) demonstrated that knee flexor peak torque was a predictor of SEBT in professional orienteering athletes. Isokinetic strength of the knee flexors was one of the six independent predictors for dominant leg and one of the nine independent predictors for non-dominant leg of the dynamic balance control (*Y*-Balance Test) in Bayesian Network analysis for futsal players (Ruiz-Pérez et al., 2019). Tasmektepligil (2016) reported significant relationship between the balance performance and isokinetic strength values at different angles for dominant and non-dominant legs among male soccer players. Our results also support earlier clinical research stressing the significance of lower extremity muscle strength for dynamic balance control. Knee flexor strength has been related with different dynamic variables of balance control, different gait, and postural variables in patients after anterior cruciate ligament (ACL) reconstruction (Domingues et al., 2018), stroke survivors (Kwong et al., 2017), and patients with multiple sclerosis (Ramari et al., 2018). One can conclude that irrespective of a subject’s age and level, hamstring muscles may play a significant role in the dynamic balance. These data support the claim that hamstring strength as an important predictor of balance control performance. Therefore, the performance achieved in the Delos Test, and consequently the dynamic balance control, appears to be widely influenced by strength flexors.

Second, it was hypothesized that that the leg’s dominance influence balance performance in unilateral stance—and the non-dominant leg as a stance leg, which supports the body—would present higher level of balance score. These results are in line with a meta-analysis which included 46 studies (Schorderet, Hilfiker & Allet, 2021). Results of this meta-analysis did not demonstrate significant differences between the dominant and the non-dominant legs in unilateral balance tests. Indeed, Paillard & Noé (2020) suggested that the possible differences in balance control between the dominant and non-dominant leg could stem from a multitude of factors other than solely neuromuscular ones (*e.g.*, morphology, proprioception, hemispheric laterality).

In soccer, the dominant leg is used with greater frequency compared with the non-dominant limb (DeLang et al., 2020). However, it should be noted that in measurement protocols of isokinetic strength and postural control various methods are used to determine the dominant and non-dominant leg, simultaneously, and there is no consensus on the optimal choice (Van Melick et al., 2017). The basis for the selection of dominant leg can be made on the basis of strength tests, functional tests (ball kick test) and footedness questionnaires (Paillard & Noé, 2020). At first, the dominant leg was defined as that used in order to manipulate an object or to lead a movement (Peters, 1988). It should be noted that the dominant leg is mainly used during performing movements that require both force and accuracy, therefore the ball kick test is a simple and reliable test which helps identify leg dominance (Paillard & Noé, 2020). Nevertheless, should be taken into consideration that other authors define the dominant leg as the stance limb initially selected when standing on one leg (Beretta et al., 2015). Thus, in cases of postural balance differences between dominant and non-dominant leg, conclusions should be used with caution.

Moreover, this present study indicates that the multivariate model revealed slightly different relationships between isokinetic knee strength and balance control in the dominant and non-dominant leg. The results suggest that apart from the largest common predictor for both legs, namely the flexor muscle, players with greater knee extensor strength showed better dynamic balance control when balancing solely on their non-dominant leg. Although the predictive effect of this muscle group on balance control is definitely smaller compared to flexor muscles (see Table 3), it can be inferred that in the non-dominant leg, greater relative knee extensor strength has the potential to improve dynamic balance control in more demanding situations (*e.g.*, single leg stance with perturbations) in comparison to the dominant leg. There is a substantial body of evidence that quadriceps muscle strength supports the knee joint during various balance tasks. A recent study by Soylu et al. (2020) also demonstrated a negative significant correlation between the overall stability index (*i.e.,* Biodex Balance System) with knee extension, and flexion peak torque of the dominant and non-dominant limb of angular velocity at 60° s^−1^ and 180° s^−1^ in female volleyball players. Soylu et al.’s (2020) results reveal that the dynamic balance performance of the athletes increased as knee extension and flexion muscle strength increased. A similar phenomenon was described by Williams et al. (2016), where stepwise backward-regression analysis included knee extension/flexion strength, among others, as a significant predictor of dynamic balance control (*i.e.,* single-leg jump landing) in a group of male soldiers. However, it is noteworthy that the protocol of this study included only measurements of the dominant leg. Moreover, some studies note only relations between knee extensors and balance control. For example Booysen, Gradidge & Watson (2015) found that that there was a moderate relationship between dynamic balance performance (*i.e., Y*-Balance Test) using the non-dominant leg for stance and eccentric strength of the non-dominant leg knee extensors in university footballers (concentric strength was not studied). This concurs with the previous studies of Lockie et al. (2013), which demonstrated that healthy male university team sport athletes with better dynamic stability (*i.e., Y*-Balance Test) also tended to exhibit greater leg strength by producing greater relative knee extensor torque at the tested isokinetic speeds. These findings are in accordance with Çelenk et al. (2015), who found that quadriceps muscle strength had a positive relationship with balance performance in 16 elite male athletes, especially in dynamic balance performance (*i.e.,* Biodex Balance System) with increasing difficulty level. Furthermore, a number of clinical data investigations support the theoretical importance of quadriceps muscle strength in supporting the knee joint during various balance tasks. In this context, research has shown that extensor strength correlates with better dynamic balance control in older cohorts with functional limitations (Moxley Scarborough, Krebs & Harris, 1999) and with Parkinson’s disease (Nocera et al., 2010). Unfortunately, there is no clear answer as to what the effect of specific knee joint muscle groups on balance control performance is, and what the relationships and proportions of these muscle groups between legs are. A possible explanation for the discrepancy in the predictors of balance control between legs in the present study may lie in the uniqueness of soccer referred to above, and the functional requirements causing specific postural adaptations within individual legs. The non-dominant leg exhibited greater sensitivity of balance control with regard to the dominant leg, which is manifested in increased activity with respect to both flexor and extensor muscles. Thus, further research is necessary to elucidate more specifically these relationships between legs in professional soccer players.

To date, there have been only two studies made available that investigated associations between balance control and knee muscle strength in professional soccer players. In the first study, López-Valenciano et al. (2019) analyzed the relationship between several parameters of neuromuscular performance with unilateral dynamic balance measured through the *Y*-Balance test. The authors reported no significant correlation between concentric knee muscle strength and balance control. These findings are in line with a study of Booysen, Gradidge & Watson (2015) who reported that eccentric isokinetic strength of the knee flexors and extensors also showed no significant impact on the *Y*-Balance test scores. Contrary to our results, these studies both indicated that concentric and eccentric isokinetic knee muscle strength does not appear to impact upon dynamic balance performance in the *Y*-Balance Test in professional soccer players. Conflicting results may arise from differences in methods of measurement of balance control (Delos Test *vs. Y*-Balance Test). Compared to the present balance control test (*i.e.,* carried out with the Delos Postural Proprioceptive System), the Y-Balance test (used for the two previous studies) involves different tasks to be undertaken that require different neuromuscular control mechanisms and strategies. Therefore, the different regulatory factors of body balance cannot be considered as invariable whatever the postural task considered (Pau et al., 2014). In fact, each balance task would differently and subtlety imply these regulatory factors.

The main limitations of the current study pertain to the analysis, which refers only to relationships between dynamic balance control and isokinetic strength for the knee joint. Moreover, it only evaluates muscle strength for concentric muscle contraction variables. Furthermore, with regard to the methodology of our measurements of balance control based on one plane of platform deflection in the sagittal axis, the measurement of isokinetic strength of adductors would be also advisable. Further research should also focus on multifaceted relationships of various elements of the whole kinematic chain during perturbed single-leg stance, *i.e.,* from the ankle, knee, and hip joint.

## Conclusion

This is the first study that has analyzed associations between concentric isokinetic strength performance and balance control during measurements by the Delos Test in elite soccer players. The final model included peak hamstrings torque and peak quadriceps torque for the non-dominant leg, and only peak hamstrings torque for the dominant leg as significant predictors of balance control during single-leg stance. The findings indicate that, depending on the leg, the performance achieved in the Delos Test, and consequently balance control, appears to be widely influenced by peak hamstring torque and peak quadriceps torque, although it worth clarifying that the predictive effect of extensor muscle group on balance control is markedly smaller compared to flexor muscles. It should also be noted that the magnitude of this contribution was greater in the non-dominant leg, albeit the mechanisms of this remain unknown. It appears that the non-dominant leg required greater sensitivity of balance control in comparison to the dominant leg, which is manifested in an increased activity with respect to both flexor and extensor muscles. Given these findings, we therefore believe that preventive training interventions should be directed towards maintaining an appropriate balance between knee extensors and flexors, mainly by strengthening the hamstring muscle in both legs (regardless of the reduced risk of muscle injury in the case of imbalance between the quadriceps and the hamstrings, since, naturally quadriceps femoris is always stronger than hamstrings, Correia et al., 2020). Likewise, these training interventions should also independently include balance exercises with altered sensory conditions (for each sensory system, *i.e.,* visual, somatosensory, and balance). Lastly, the development of multiple other neuromuscular factors would help to better control monopedal balance in male soccer players and thus further research is necessary to better ascertain the relationships between knee extensor-flexor strength and balance control.

## Supplemental Information

10.7717/peerj.12461/supp-1 Supplemental Information 1 Raw data All measurements that were used for the statistical analysis.

## Additional Information and Declarations

### Data Availability

The following information was supplied regarding data availability: The raw data are available in the Supplementary File.
